# Effect of Somatic Experiencing Resiliency-Based Trauma Treatment Training on Quality of Life and Psychological Health as Potential Markers of Resilience in Treating Professionals

**DOI:** 10.3389/fnins.2018.00070

**Published:** 2018-02-16

**Authors:** Neal E. Winblad, Michael Changaris, Phyllis K. Stein

**Affiliations:** ^1^Private Pracitce Truama and Developmental Trauma Specialist, Pleasanton, CA, United States; ^2^Health Psychology Department, Integrated Health Psychology Training Program, The Wright Institute, Berkeley, CA, United States; ^3^Heart Rate Variability Laboratory, Cardiovascular Division John T. Milliken Department of Medicine, School of Medicine, Washington University in St. Louis, St. Louis, MO, United States

**Keywords:** Somatic Experiencing®, resiliency, vicarious traumatization, compassion fatigue, burnout, quality of life, ANS dysregulation, traumatic stress

## Abstract

**Background:** Individuals who treat trauma are at significant risk of vicarious traumatization and burnout. Somatic Experiencing® (SE®) is a resiliency-focused trauma treatment modality designed to address autonomic nervous system (ANS) dysregulation and its impacted physical health and mental health symptoms e.g., anxiety, depression, post-traumatic stress disorder, migraines, fibromyalgia, and chronic fatigue, etc. The SE® training supports the development of clinical skills to reduce physical health/mental health symptoms as well as increase clinician resilience. Individuals who display resilience often have increased experiences of well-being (quality of life) and decreased levels of self-reported psychological symptoms. Greater resilience could mitigate the risks to providers and the clients they treat.

**Materials and Methods:** This within-groups, longitudinal study assessed students (*N* = 18) over the course of a 3-year SE® practitioner training. This training focuses on increased ANS, physical, and emotional regulation skills. The convenience of a web-based survey allowed for: measures of a general quality of life (WHOQOL-BREF), psychological symptoms, somatic, anxiety, and depressive symptoms (PHQ-SADS), as well as a measure of early life exposure to adversity (CDC/Kaiser Permanente ACE Score Calculator Questionnaire). The clinician survey was conducted yearly for 3 years. Future studies would do well to also include laboratory-based objective measures of ANS functioning.

**Results:** ANOVA with repeated measures showed that there were significant reductions in anxiety symptoms (GAD7, *p* < 0.001) and somatization symptoms (PHQ15, *p* < 0.001). Health-related quality of life (a measure of physical well-being) and social quality of life (a measure of interpersonal well-being) both increased significantly (Health QoL *p* = 0.028; Social QoL *p* = 0.046).

**Conclusions:** Results suggest that professionals attending the 3-year SE® training course experience a significant improvement in self-reported measures associated with resiliency including: quality of life (well-being) and psychological symptoms (anxiety and somatization). Our results support the importance of future research in a larger sample and support the exploration, cross-sectionally and prospectively, of the relationship of clinician resiliency and changes in clinician resiliency with SE® training and clinical outcomes. These data have implications for other professions at risk of exposure to vicarious trauma (VT) including nurses, medical providers, and paramedics.

## Introduction

Individuals who treat traumatized and other highly dysregulated clients are at higher risk of developing symptoms of burnout, compassion fatigue and vicarious traumatization/secondary trauma (Sprang et al., 2007; Craig and Sprang, 2010). Professionals who develop these symptoms are less effective in their work, need to seek their own treatment, and can experience long-term negative health outcomes (Pearlman and Saakvitne, 1995; Baird and Jenkins, 2003; Trippany et al., 2004; Craig and Sprang, 2010). Professionals who treat PTSD may also be required to work in areas and situations that could increase their own chances of exposure to traumatic events, e.g., war zones or areas with high community violence (Johnson et al., 2014).

Richardson and Waite (2002) define resiliency as “a self-righting force within everyone that drives him/her to pursue self-actualization, altruism, wisdom, and harmony with a spiritual source of strength.” Others define resilience as the ability to adapt to adversity (Horn et al., 2016). Li et al. (2016) demonstrated a correlation between social support, hope, resilience, and quality of life in bladder cancer patients, with resilience, hope and social support accounting for 30% of the variance in quality of life. Resilience and hardiness has been found to mitigate the effects of trauma exposure, improve neuroendocrine functioning and improve coping (Tugade and Fredrickson, 2004; Ménard et al., 2016).

Clinicians who develop the capacity to manage, reduce and mitigate impacts of stress-based injuries may be more effective at treating PTSD and other symptoms of intense dysregulation in both their offices and in the field (Horn et al., 2016; Pereira et al., 2016). Somatic Experiencing® (SE) is a trauma treatment modality that works with trauma through supporting innate resilience and reducing stress, as well as stress-based injuries. A growing body of research has demonstrated improvements in multiple symptom domains as a result of SE® treatment (Leitch, 2007; Whitehouse and Heller, 2008; Andersen et al., 2017; Brom et al., 2017).

### Trauma, burnout, and vicarious trauma

Rates of burnout, secondary traumatic stress (STS) and vicarious trauma (VT) are high among treating professionals. Increased resilience, social support and coping strategies can mitigate both risk and impact of exposure (Bell et al., 2003). Secondary traumatic stress (STS) is a traumatic reaction that can be catalyzed from a single exposure to another individual's experience of a traumatic event, whereas VT is a result of cumulative exposure to traumatic experiences over time. Thus, among therapists, VT is triggered by a cumulative exposure to traumatic experiences of patients/clients and refers to harmful changes in how a professional views their work, themselves, their world and other professionals (Pearlman and Saakvitne, 1995; Baird and Jenkins, 2003). Both STS and VT can impact clinicians who work with individuals exposed to trauma and can affect their functioning and lead to an array of stress reactions.

Estimates of rates of STS among psychotherapist working with trauma in military settings are as high as 19.2% (Cieslak et al., 2013). Research into VT among nursing and medical providers has identified a prevalence of between 26 and 40% of individuals experiencing symptoms due to their work experiences. Child protective services workers, for example, were found to have a 35% rate of VT in one study (Sabin-Farrell and Turpin, 2003).

Understanding the basic principles of risk and resilience in treating professionals and developing self-regulation/emotion regulation capacity could improve functioning and reduce risk of VT (Pearlman and Saakvitne, 1995; Baird and Jenkins, 2003; Pereira et al., 2016). Risk factors for both STS and VT include a history of previous traumatic events. The temporal exposure to client traumatic experiences, e.g., caseload, hours with clients, percent of trauma clients in caseload, and amount of exposure over time has also been related to risk for both VT and STS in some studies (Pearlman and Saakvitne, 1995). Perception of coping has been found to mitigate symptoms of both VT and STS (Pearlman and Saakvitne, 1995).

While many programs aimed at increasing resilience focus on cognitive coping, a recent study found some significant limitations to top-down cognitive coping strategies. In this recent study, in the general population, cognitive regulation (using cognitive coping) alone was found to have a limited impact on cortisol, a stress hormone dysregulated in PTSD (Pereira et al., 2016). These data point to the need for the inclusion of additional non-cognitive coping capacities for effective clinician resilience training.

The costs of VT and STS are significant for the individual, the people they treat and the families of the trauma treating professional (Baird and Jenkins, 2003; Cocker and Joss, 2016). Developing strategies for improved resilience among treatment providers could help mitigate these costs.

### Clinician resilience and clinical outcomes

Many factors have been shown to be associated with clinician resilience. Some of these are: cognitive coping strategies, mindfulness, ability to access social support, and effectiveness of self-care. Mindfulness has been associated with reduced symptoms of PTSD and increased resilience in multiple populations. Fisher and Ogden (2009) developed the term “somatic mindfulness” to describe the ability to increase interoceptive awareness of bodily states and self-regulatory capacity in her work on Sensorimotor Psychotherapy™. In other research, clinician mindfulness was found to be related to better clinical outcomes. A 2016 study of mindfulness and clinician resilience found that to the degree that an individual practiced mindful self-awareness, such as meditation, they had improved clinical outcomes (Pereira et al., 2016). The Pereira study reviewed 37 therapists' caseloads for a total of 4,980 cases. A significant proportion of the clinician/therapist factor in effective clinicians was due to mindfulness and resilience. These data indicated that for less severe cases (mild to moderate depression) mindfulness alone improved clinical outcomes, but in the more severe cases (severe depression) the combination of mindfulness and resilience affected outcomes. In somatic therapies, such as Somatic Experiencing® and Sensorimotor Psychotherapy™, both increased clinician resilience and somatic mindfulness are core parts of training and interventions.

### Somatic experiencing® and resilience

Somatic Experiencing® (SE®) is a resiliency-based approach to trauma treatment that, rather than focusing on pathology, focuses on working with innate resilience and increasing capacity (Payne et al., 2015). SE® uses a bottom up approach that works with interoceptive awareness, affective states, and limbic activation. SE® differs from many cognitive therapies which work to change cognitions in order to change affective states. SE® interventions are designed to help guide the client to increased contact with their bodily sensations (interoceptive cues and kinesthetic/muscular awareness), instead of focusing on cognitions. This bottom up approach is based on the fact that core aspects of trauma are housed in systems which emerge from brain structures deep below the cortex. As van der Kolk (1984) showed, in his initial studies of traumatized patients, central language centers of the brain (i.e., Brocca's area) are often shut down during reactions to trauma triggers. As a result, there is some significant aspect of the traumatic experience that is housed in what van der Kolk describes as “wordless terror” (van der Kolk, 1984).

Somatic Experiencing® works with what Payne et al. (2015) paper call the Core Response Network (CRN). The CRN includes subcortical, limbic system, motoric pathways, interoceptive cues, and basic arousal systems [i.e., the autonomic nervous system (ANS), the hypothalamic-pituitary-adrenal axis and the reticular activating system]. Dysregulation in these protective systems leads to the development of symptoms of trauma and other negative health outcomes. The SE® modality works by supporting the re-establishment of the innate regulatory capacity of the CRN via interoception (a core component of mindfulness), self-protection, emotion regulation, and self-awareness (Payne et al., 2015). To support the goal of re-establishing innate regulatory capacity (e.g., resilience), interventions are conducted in a client-centered, titrated manner.

While increased awareness and tolerance for affective states is a core outcome of SE® therapy, it is not primarily an exposure therapy. Rather than evoking intense traumatic memories directly, SE® works indirectly and gradually with these memories, identifying resources and corrective shifts in states that lead to new interoceptive experiences that in turn lead to changes in the felt experience of safety, power, and competence. While SE® shares many components with traditions such as meditation, Qigong, and yoga, it also has a specific set of tools that help clinicians and clients address the fundamental dysregulation of the CRN that underlies trauma (Payne et al., 2015). Parker et al. (2008) showed that tsunami victims in southern India showed 90% improvement in symptoms even at eight month follow up to short 75 minute treatment sessions using SE_R_ skills.

### Somatic experiencing® training and resilience

The Somatic Experiencing® Practitioner training course is open to all practitioners who come into contact with clients struggling with the symptoms of trauma. It has three beginning modules (4 days each), three intermediate modules (4 days each), and two advanced modules (6 days each). In the beginning year students develop, through practice, their own resilience and are encouraged to practice self-regulation throughout the training. Students also learn the basic biology of trauma and resilience, core clinical skills for guiding and improving interoceptive awareness and integration of protective strategies mobilized to protect the body during a traumatic event. In the intermediate year students learn to address specific types of trauma, e.g., falls, motor vehicle accidents, natural disasters, violence, etc. In the advanced year, students learn skills for working with the biological syndromes that are often comorbid with trauma such as fibromyalgia, migraines, and IBS. Throughout the training and between training modules, participants are encouraged to practice self-regulation skills, work in consultation groups, and have regular sessions that support their own development of self-regulation capacity and increase their skill as trauma treating therapists. They are also encouraged to read and explore the literature of trauma, trauma healing, and self-regulation. It is expected that in addition to becoming skillful as therapists in treating trauma, these practices will lead to the increased ability to self-monitor, regulate affect, cope with stress, stay in mindful awareness, and improved resilience, although ours is the first study to explicitly test this hypothesis.

### Aim

While the most traditional definition of resilience is the ability to withstand and rebound from adversity, in a study involving human subjects it would be unethical to create severe adversity in subjects lives in order to study their immunity to adversity. We therefore looked at secondary indicators of resilience in this study, which can be measured on self-report psychological measurement instruments.

The purpose of this study was to test the effect of Somatic Experiencing® Training on measures of resiliency by assessing changes in clinician resilience longitudinally over the 3 year SE® training course. Clinician quality of life, psychological symptoms and degree of early life trauma were measured. We used the World Health Organization Quality of Life-Brief (WHOQOL-BREF) to assess changes in well-being in four domains: psychological quality of life (mental wellness), health related quality of life (overall physical well-being), social quality of life (quality of social relationships), and environmental quality of life (measure of wellness on community and access to housing etc.,). Changes in psychological symptoms was measured using the Patient Health Questionnaire (PHQ-SADS), which includes three subscales for anxiety (GAD-7), somatic symptoms (PHQ-15), and depression (PHQ-9). In order to assess the early life adversity on changes in resilience, early life adversity was measured using the CDC/Kaiser Permanente ACE^1^ Score Calculator Questionnaire.

## Methods

### Recruitment

Due to multiple challenges with sampling and randomization, a convenience sample of volunteer participants in the SE® 3-year training course was used. Subjects were recruited from the cohort of students participating in the Somatic Experiencing® training course at five different sites: Berkeley, CA, Columbus, OH, Chapel Hill, NC, NYC, NY, and Austin, TX. Individuals who attend the Somatic Experiencing® training course are an interdisciplinary group of health care providers including: psychologists, social workers, medical doctors, psychiatrists, physical therapists, and other body oriented therapists. During the Beginning I module of the SE® training course, the lead teaching assistant read a recruitment script for the experimental group. Potential subjects volunteered by signing up and the lists of volunteers were sent to the principal investigator.

### Experimental design

**Figure 1:**
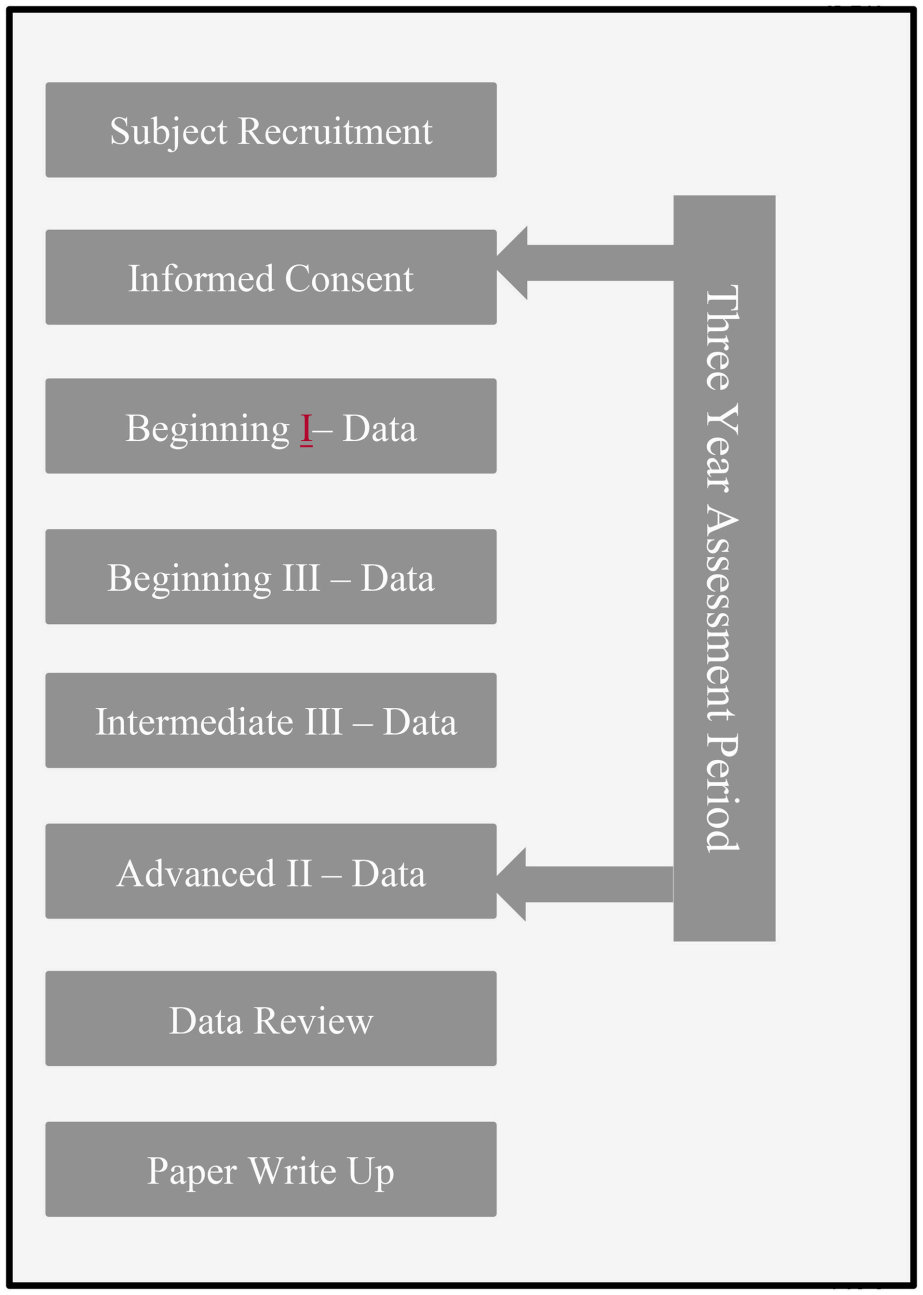
Study timeline. This figure displays the study timeline including informed consent and data collection points. Data was collected over the course of the 3-year training period.

This study was a within-groups pre-to post-test design. The within-groups methodology and multiple points of measurement reduce some of the variance found in between-groups designs. Study volunteers were assessed on changes over the course of 3 years on four variables: quality of life, physical symptoms, psychological symptoms, and at the initial measurement the effect of adverse childhood experiences. Beginning and intermediate training modules are 4 days (24 h in length), the advanced training year modules are 6 days (36 h of continuing education). As shown in Figure 1, there were four points of measurement: at the start of SE® training course (beginning I), after the beginning year (end of beginning III, after the intermediate year (end of intermediate III), and after completion of the training course (after year three advanced level II).

### Outcome measures

#### WHOQOL-BREF

The WHOQOL-BREF has four quality of life domains: psychological quality of life (PSYCH-QOL or mental wellness), health related quality of life (HEALTH-QOL or overall physical well-being), social quality of life (SOCIAL-QOL or quality of social relationships), and environmental quality of life (ENVIRONMENTAL–QOL or measure of wellness on community and access to housing etc.) (The WHOQOL Group, 1995; Power, 1998).

#### PHQ-SADS

The PHQ-SADS is a validated measure of stress, anxiety, somatic symptoms, and depression. (Han et al., 2009; Kroenke et al., 2010a,b; Kocalevent et al., 2013). The PHQ-SADS includes a question about distress and three subscales measuring anxiety (GAD-7), somatic symptoms (PHQ-15), and depression (PHQ-9).

### Data collection

Participants were contacted by email and given the URL for the web-based survey built around Limesurvey open source software (Engard, 2009). The survey included a short algorithm for calculating a subject identification number, such that the online data were de-identified before data entry and results were completely de-identified.

The web survey site asked participants to agree to the terms and conditions after reading the recruitment statement. It then collected some generic demographic information, followed by the survey questions. Participants were asked to send an email to the principal investigator acknowledging that they had completed the survey and informing him which cohort they were a part of. The PI tracked the training module schedule for each participant, and 2 weeks after the relevant modules, the PI sent emails to each study participant informing them that it was time to retake the survey. The participants would then take the survey and send an email to the PI announcing that they had completed this round of the survey. If no response was received a second email notice was sent.

### Statistical methods

ANOVA with repeated measures was used to test the hypotheses that students who completed the 3 years SE® training would show an increase in each of the four domain measures of quality of life measured using the WHOQOL BREF during the training and would also have decreases in symptoms of anxiety and somatic symptoms compared to baseline (PHQ-SADS) during the course of the training. *Post-hoc* analysis used Fischer's least significant difference (LSD) to assess differences between periods of measurement (i.e., years of the study) and changes in specific variables. Software was SPSS 23.0. Statistical significance was set at *p* = 0.05.

## Results

### Participants

*N* = 16 participants self-identified as female and *N* = 2 as male. *N* = 12 participants self-identified as middle class and *N* = 5 as upper class. None reported being lower SES.

### Participant flow

Initially *N* = 45 participants signed up. After completion of the training, *N* = 18 remained in the study. Per participant report, dropout was largely due to attrition in the training for financial reasons, needing to postpone finishing the training, loss of interest in SE®, and no longer wishing to participate in the survey.

### Adverse events

To date there have been multiple studies exploring SE's impact on PTSD and other health/mental health challenges published in peer reviewed journals (Heller and Heller, 2004; Leitch, 2007; Leitch et al., 2009; Changaris, 2010). No significant adverse events were reported as a result of conducting these studies in any of these and none occurred in the current study.

### Detailed results

#### WHOQOL-BREF

**Figure 2:**
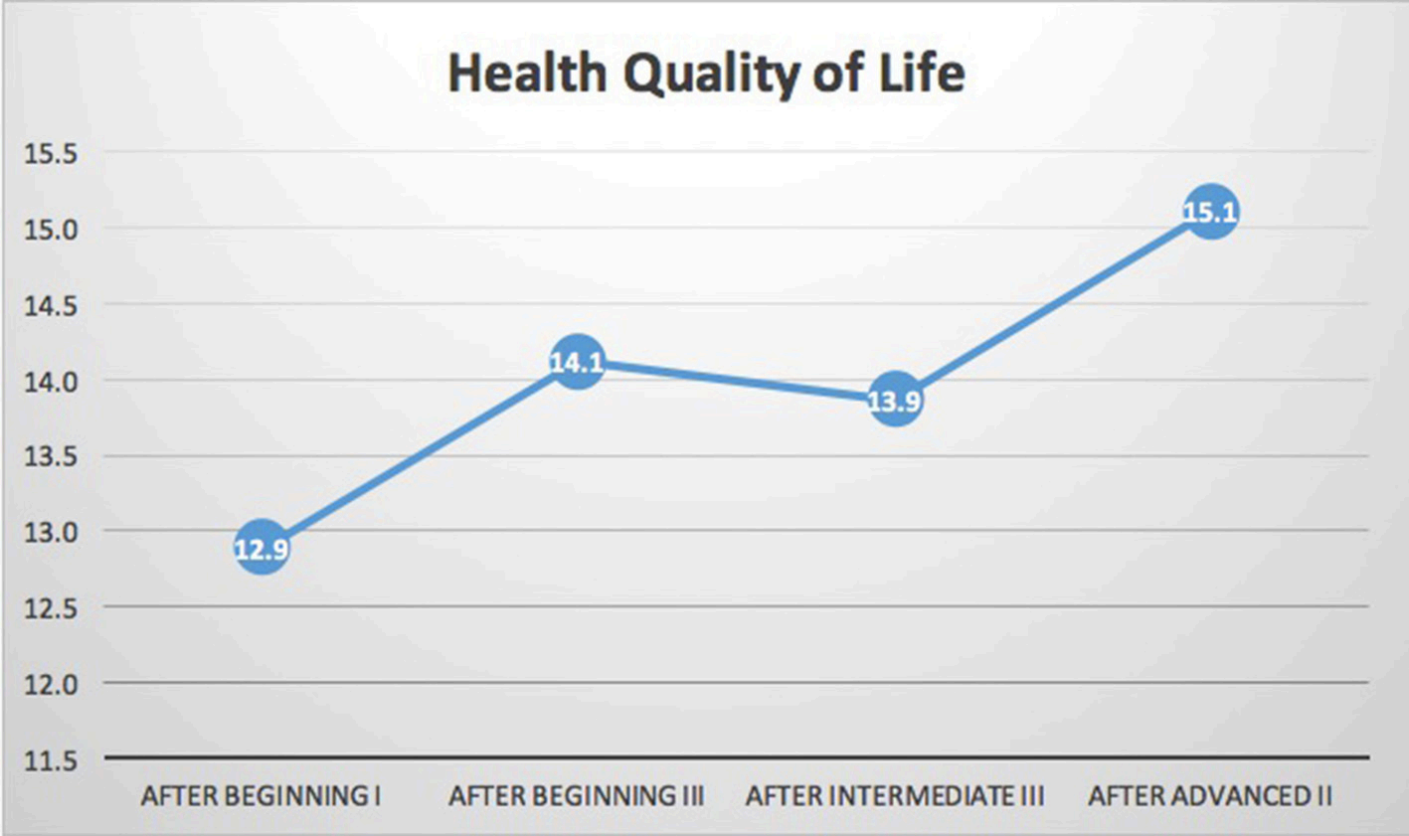
WHOQOL-BREF health related quality of life. Results indicated significant improvement in health related quality of life (*p* = 0.028) on the WHOQOL-BREF. While the sample size is too small to clearly identify trends it is notable that there was a flattening of the trend line during the intermediate training year i.e., between the final beginning class (Beginning III) and final intermediate class (Intermediate III). Intermediate year training focuses on addressing specific types of traumas and focuses less on training of emotional regulation skills. It is possible that the exposure to learning about specific traumatic events may lead to a short-term level of distress that is reduced in the final two advanced year trainings.

**Figure 3:**
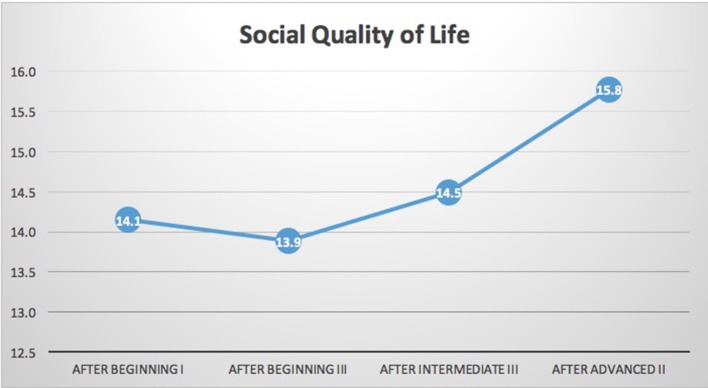
WHOQOL-BREF social quality of life. Results indicated significant improvement in social quality of life (*p* = 0.046) on the WHOQOL-BREF. The trend in social quality of life was flat over the course of the three beginning modules. As above the sample size is too small to clearly identify trends. The beginning training year has several aspects that would address social quality of life. One of the core modules that could impact social quality of life is somatic boundaries and trauma. This module focuses on interpersonal safety and setting limits to support self-regulation. It could be that these skills require more significant practice before they can impact one's social connections or social quality of life. Alternatively, social quality of life may have a lag time from skill increase to change in behavior due to relationships being built over time.

*N* = 15 participants had complete data for the WHOQoL-BREF. As shown in Table 1 and Figures 2, 3, significant improvements in quality of life measures were observed in two of the four domains of the WHOQoL-BREF, Health-QoL (*p* = 0.028), and Social-QoL (*p* = 0.046). Health improved significantly between the initial measurement and each of three subsequent measurements. Social-QOL increased significantly at the fourth measurement compared to each of the prior measurements. There were changes in Psychological and Environmental QoL but analysis showed them not to be significant.

**Table 1:** Changes in Quality of Life (QOL) as Measured by the WHOQOL-BREF (*N* = 15).

	**Beginning I**	**Beginning III**	**Intermediate III**	**Advanced II**	**p for model**
HEALTH-QOL	15.4 ± 2.4	16.2 ± 0.1^*^	16.5 ± 2.1^*^	16.3 ± 2.4^*^	0.028
PSYCH.-QOL	14.4 ± 2.0	14.5 ± 2.5	15.3 ± 2.2	15.6 ± 2.0	NS
SOCIAL-QOL	13.5 ± 4.1^**^	13.4 ± 3.4^**^	13.9 ± 3.5^**^	15.7 ± 2.8	0.046
ENVIRONMENTAL-QOL	15.5 ± 2.5	15.7 ± 2.7	16.2 ± 2.1	16.4 ± 1.9	NS

* *Significantly increased compared to Beginning I, p ≤ 0.033*.

** *Significantly lower compared to Advanced II, p ≤ 0.025*.

#### PHQ-SADS

**Figure 4:**
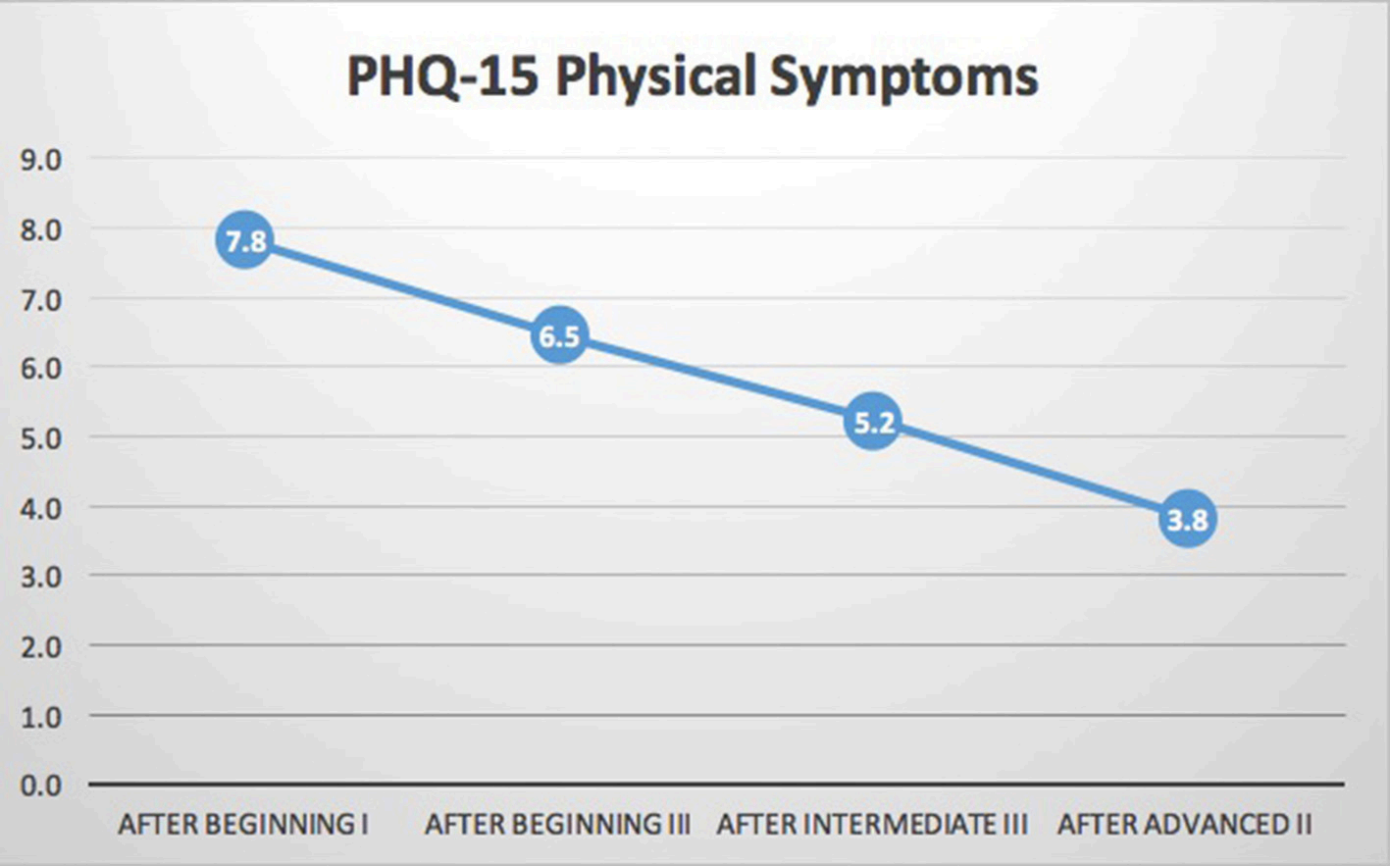
PHQ-15 somatic symptoms. Results indicated significant reductions in somatic symptoms scale (PHQ-15) of the PHQ-SADS (*p* < 0.001). While the data set is too small for clear trends to emerge, these data indicate a possible step-wise reduction in symptoms over the course of the training. The main focus of the training in each module is increased somatic awareness, capacity to regulate stress states when engaging in clinical work and increased skills in recognizing one's own indication of stress states through interoception and description of the state. The regular time for reflection and skills practice could be a driver for the change at each point of measurement.

**Figure 5:**
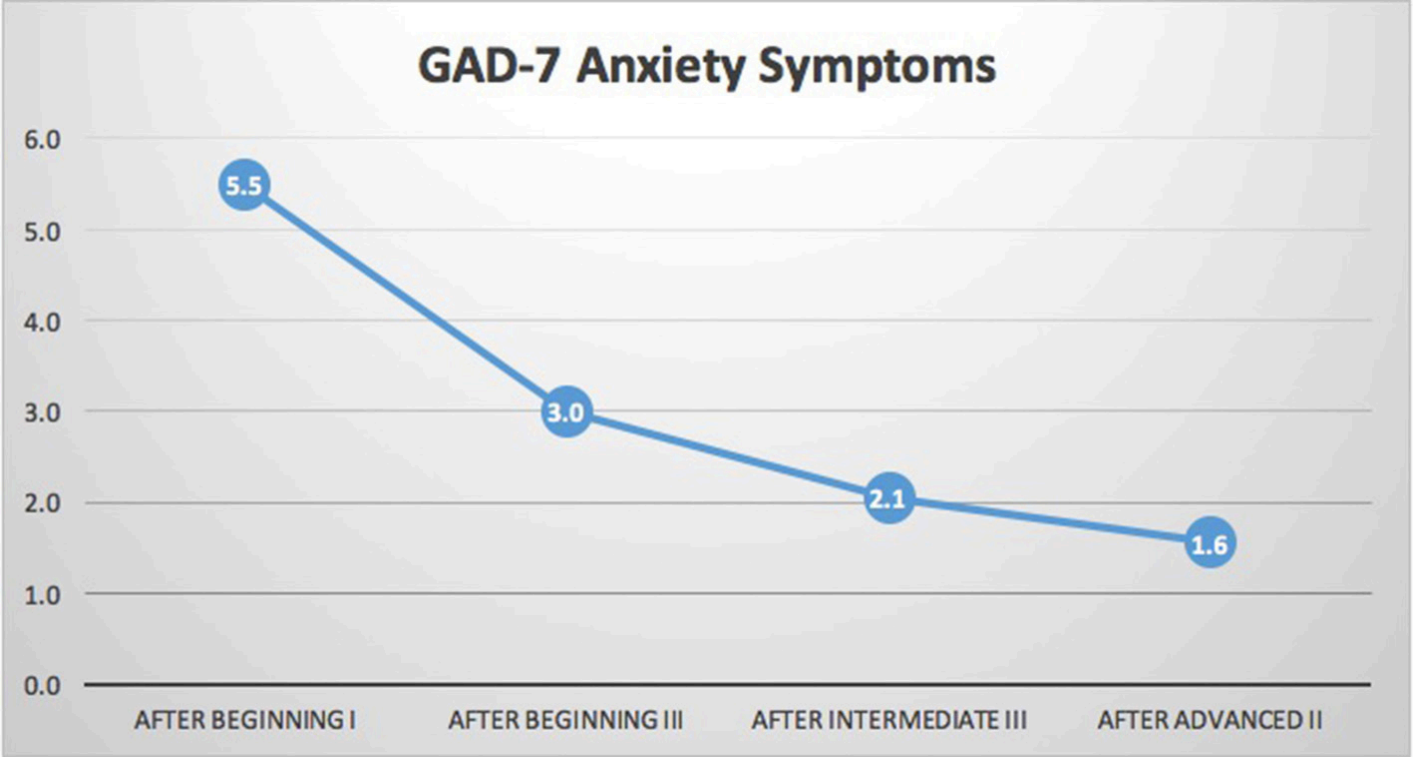
GAD-7 generalized anxiety scale. Results indicated significant reductions in anxiety scale (GAD-7) of the PHQ-SADS (*p* < 0.001). As stated above, the data set is too small for trends to be clearly identified but there appears to be a stable downward trend in symptoms of generalized anxiety. There is a slight flattening of the downward trend in the last two points of measure. It is likely that there are floor effects as the symptom level trends toward zero in the last two measures.

*N* = 18 participants had complete data for the PHQ-SADS. As shown in Table 2 and Figures 4, 5, significant reduction in Anxiety (GAD-7) and Somatic (PHQ-15) symptoms was observed (*p* ≤ 0.001 for each). In general, scores improved between each training year. In *post-hoc* analysis, all periods of measurement were significantly different between first and follow up measures. The only non-significant changes were between the third (intermediate III) and fourth (advanced II) measurements. The PHQ-SADS also includes a single question that measures overall distress from the anxiety, somatic, and depression symptoms reported. Scores on the overall symptom distress question were significantly reduced between initial measurement (post beginning I) and final measure (post advanced II) training years (*p* < 0.001). Depression, as measured by PHQ9, could not be assessed due to an error in the question design in the survey platform leading to insufficient data.

**Table 2:** Changes in PHQ-15 and GAD-7 as Measured by the PHQ-SADS (*N* = 18).

	**Beginning I**	**Beginning III**	**Intermediate III**	**Advanced II**	**p for model**
PHQ-15 (Som.)	7.8 ± 3.1	6.1 ± 3.1^*^	4.9 ± 3.5^*^	3.8 ± 2.4^*^	<0.001
GAD-7 (Anx.)	5.5 ± 4.6	3 ± 3.2^*^	2 ± 2.3^*^	1.5 ± 2.4^*^	<0.001

* *Significantly decreased compared to Beginning I, p ≤ 0.013. NS for Intermediate III vs. Advanced II*.

### PHQ-SADS graphic display of data

#### ACE scores

ACE scores were available at each time point for 15 participants. Self-reported ACE scores were not significantly different over the four assessments. No relationship was found between any of the measurements at Beginning I and ACE scores, due likely to the small sample size and the small number of subjects with high ACE scores.

## Discussion

Rates of community exposure to trauma are extremely high, in some cases > 60% of the population (Felitti et al., 1998; Anda et al., 2009). This has caused leaders in the field to call trauma the “hidden epidemic of our era.” The ability of health practitioners to address symptoms of trauma in their health and mental health care systems could have a profound impact on multiple chronic diseases (e.g., diabetes, hypertension, chronic pain, addiction, and stroke). However, trauma treatment also *exposes* individual professionals to the risk of vicarious traumatization, compassion fatigue and health related stress conditions. Some studies have indicated that compassion fatigue can lead to adverse changes in inflammatory cytokines and metabolic functioning and thereby increase risk for multiple negative health outcomes (Huffman, 2016). These health conditions also can impact treatment outcomes, as well as result in a significant burden on the healthcare system in the form of mental health worker attrition and of mental health workers who may become the “walking wounded.” These treatment providers continue to show up to their jobs, but, due to the burden of stress, are compromised in their ability to actually get their jobs done.

A 2017 randomized study of Somatic Experiencing in individuals with PTSD trauma found significant reductions across multiple domains (Brom et al., 2017). One of the foundational concepts in the SE® model is that the increased clinical resilience is a likely outcome of training in the SE® model (Levine, 2010; Payne et al., 2015). Significant time and focus of the SE® training is directed toward increased clinician resilience. Further, the SE® model would indicate that increased clinician resilience would likely impact clinical outcomes (Levine, 2010). This study is the first study to explore the impact of training in the SE® model on clinician resilience. Future research could explore issues of clinician case load and the question of impact of clinician resilience on clinical outcomes.

Another 2017 randomized control study of the SE® model was conducted with 91 pain patients found to have PTSD (Andersen et al., 2017). This study noted significant reduction of trauma symptoms and fear of movement as compared to controls. Patients with chronic pain have significantly higher rates of trauma than the general population. Physicians who treat pain patients report high levels of burnout and fatigue (Brennstuhl et al., 2015; Kroll and Macaulay, 2016). While the scope of this present study focuses on behavioral health professionals, increased clinician resilience in providers who treat pain patients could have a meaningful impact on current pain management teams (Kroll and Macaulay, 2016).

Results of the current study indicate that among individuals who completed the SE® training, there were significant reductions in psychological symptoms of anxiety, and also in somatic symptoms. Participants in the training showed an ongoing reduction in symptoms over the course of the three training years. This reduction was both statistically significant and clinically meaningful with both somatization symptoms and symptoms of generalized anxiety dropping from mild to well below a clinical range. Notably, this was occurring at the same time that many of these individuals were treating individuals with symptoms of trauma and also working with SE® professionals on their own trauma histories. Results also indicate a significant increase in health-related and social quality of life among trainees. While both psychological quality of life and physical health related quality of life trended in a positive direction neither reached significance in this cohort. Further studies could elucidate the patterns driving these outcomes.

Psychological quality of life also appears to have increased at the final measurement compared to baseline. However, the change in psychological quality of life was not significant. Of course, improvement in psychological quality of life correlates with changes in psychological symptoms such as the ones found in this study. One possible moderating factor in this change is that the GAD-7 and PHQ-15 are measures of more specific domains likely to be impacted by the training in somatic self-regulation but the psychological quality of life measure used in this study was broader and less targeted to this outcome. Another possible driver could simply be that for this measure the study lacked the needed sample size for the effect size on this measure and a larger sample size could have more power and thus be more sensitive to the effects on psychological quality of life. Results of this pilot study support the importance of further study and assessment of real-world impacts, both on clinical outcomes and long-term clinician resilience, among professionals who treat trauma and trauma related conditions.

## Limitations

The current study's design limits its generalizability. This is a small convenience sample of professionals and no control group. A possible enhancement would be to include a comparison group consisting of other professionals engaged in some other type of trauma training and a control group of professionals who have not had any form of trauma training. Another limitation impacting broader generalization of this study's findings was lack of data on the clinical setting of students in the study and their caseloads. An enhancement for future studies would be to include questions related to practice context and caseload to compare with changes in measures.

The current study had a problem with a high dropout rate. This is for several reasons: (1) no compensation of any sort was offered to the participants, (2) some students were forced to drop out of the training due to lack of funds. This is especially true for graduate students and interns. (3) some students, due to scheduling conflicts and lack of paid time off, are forced to wait an extra year to complete their SE® training, (4) some students lose interest in studying trauma, and (5) the mathematical algorithm which needed to be calculated by participants in order to de-identify themselves proved to be daunting for some participants. Starting with a larger sample size and offering some incentive, as well as making the de-identification process easier would all help with this issue.

## Directions for future research

Increased clinician resilience has profound implications for multiple professions and health/mental health providers. Future research would involve replicating these results with a larger sample size.

Other possibilities for future research would be using a control group of trauma therapists who do not undergo SE® training, and examining clinician resilience vs. client outcomes. The impact of resiliency training on other high-risk profession groups such as substance abuse and suicide prevention counselors should be explored. Further research should collect data on the clinical setting of treating providers, caseloads and number of trauma patients.

Establishing a more direct causal pathway for these findings (reduction of anxiety, physical symptoms, and improvement in quality of life) with respect to resiliency, work satisfaction, and thus therapist retention within the trauma treatment field, would be a useful contribution to this area of study.

Resiliency is a complex heuristic that is still being clarified as to its scope. While further research is needed to confirm these findings, it is also possible, in future studies, to dissimilate the core components of increased resilience identified in this study. Researches exploring specific aspects of the training that mediate and moderate changes in resiliency could further support the field in helping to increase resilience, reduce the psychological impacts of trauma treatment, and improve outcomes in both therapists and clients. The measures used in this study are global measures of psychological health. There are multiple possible drivers for the changes identified in this study. While it was beyond the scope of this study to assess possible mechanisms of change the authors identified five candidates for future research. These are: (1) increased somatic mindfulness due to greater interoceptive awareness (Payne et al., 2015; Haase et al., 2016), (2) increased skills at resourcing or evoking a parasympathetic/calming response (Park et al., 2013), (3) increased acceptance and tolerance for intense sympathetically-mediated arousal states (Thompson et al., 2011; Nila et al., 2016), (4) changes in biological markers of sympathetic activation, ANS regulation and physiological health indicators (e.g., blood pressure, inflammatory cytokines or 24 h salivary cortisol), and (5) increased efficacy in clinicians, due to increased skills in addressing client symptoms (Shoji et al., 2016).

## Ethics statement

This study involved human subjects and was supervised by the private IRB, *Independent, and Ethical Review Services* under case number: 14033–03. All consent and data collection procedures were conducted in accordance with Declaration of Helsinki and guidelines outlined by IRB committee.

## Author contributions

NW: Principal Investigator; NW, MC, and PS: Conceived and designed the study; NW: Fundraising; NW: IRB Interface; NW: Performed the study; NW, PS, and MC: Analyzed the data; NW, MC, and PS: Wrote the paper; NW, MC, and PS: Response to reviewers.

### Conflict of interest statement

The authors declare that the research was conducted in the absence of any commercial or financial relationships that could be construed as a potential conflict of interest.

## Footnotes

^1^Centers for Disease Control and Prevention: Adverse Childhood Experiences (ACE) Study. Available online at: http://www.cdc.gov/ace/index.htm

## References

[B1] Anda R. F. Dong M. Brown D. Felitti V. Gile W.. (2009). Adverse childhood experiences and the risk of premature mortality. J. Prev. Med. 37, 389–396. 10.1016/j.amepre.2009.06.021 19840693

[B2] Andersen T. E. Lahav Y. Ellegaard H. Manniche C. (2017). A randomized controlled trial of brief Somatic Experiencing for chronic low back pain and comorbid post-traumatic stress disorder symptoms. Eur. J. Psychotraumatol. 8:1331108. 10.1080/20008198.2017.1331108 28680540 PMC5489867

[B3] Baird S. Jenkins S. R. (2003). Vicarious traumatization, secondary traumatic stress, and burnout in sexual assault and domestic violence agency staff. Violence Vict. 18, 71–86. 10.1891/vivi.2003.18.1.71 12733620

[B4] Bell H. Kulkarni S. Dalton L. (2003). Organizational prevention of vicarious trauma. Famil. Soc. J. Contemp. Soc. Ser. 84, 463–470. 10.1606/1044-3894.131

[B5] Brennstuhl M. J. Tarquinio C. Montel S. (2015). Chronic pain and PTSD: evolving views on their comorbidity. Perspect. Psychiatr. Care 51, 295–304. 10.1111/ppc.12093 25420926

[B6] Brom D. Stokar Y. Lawi C. Nuriel-Porat V. Ziv Y. Lerner K.. (2017). Somatic experiencing for posttraumatic stress disorder: a randomized controlled outcome study. J. Trauma. Stress 30, 304–312. 10.1002/jts.22189 28585761 PMC5518443

[B7] Changaris M. C. (2010). Assessing the Efficacy of Somatic Experiencing for Reducing Symptoms of Anxiety and Depresssion, Doctoral dissertation, John F. Kennedy University.

[B8] Cieslak R. Anderson V. Bock J. Moore B. A. Peterson A. L. Benight C. C. (2013). Secondary traumatic stress among mental health providers working with the military: prevalence and its work-and exposure-related correlates. J. Nerv. Ment. Dis. 201, 917–925. 10.1097/NMD.0000000000000034 24177477 PMC4892748

[B9] Cocker F. Joss N. (2016). Compassion fatigue among healthcare, emergency and community service workers: a systematic review. Int. J. Environ. Res. Public Health 13:618. 10.3390/ijerph13060618 27338436 PMC4924075

[B10] Craig C. D. Sprang G. (2010). Compassion satisfaction, compassion fatigue, and burnout in a national sample of trauma treatment therapists. Anxiety Stress Coping 23, 319–339. 10.1080/10615800903085818 19590994

[B11] Engard N. C. (2009). LimeSurvey. Available online at: http://limesurvey.org

[B12] Felitti V. J. Anda R. F. Nordenberg D. Williamson D. F. Spitz A. M. Edwards V.. (1998). Relationship of childhood abuse and household dysfunction to many of the leading causes of death in adults. The Adverse Childhood Experiences (ACE) study. Am. J. Prev. Med. 14, 245–258. 10.1016/S0749-3797(98)00017-8 9635069

[B13] Fisher J. Ogden P. (2009). Sensorimotor psychotherapy, in Treating Complex Traumatic Stress Disorders, An Evidence Based Guide, eds Courtois C. A. Ford J. D. (New York, NY: Guilford Press), 312–328.

[B14] Haase L. Stewart J. L. Youssef B. May A. C. Isakovic S. Simmons A. N. (2016). When the brain does not adequately feel the body: links between low resilience and interoception. Biol. Psychol. 113, 37–45. 10.1016/j.biopsycho.2015.11.004 26607442 PMC6559799

[B15] Han C. Pae C. U. Patkar A. A. Masand P. S. Kim K. W. Joe S. H.. (2009). Psychometric properties of the Patient Health Questionnaire-15 (PHQ-15) for measuring the somatic symptoms of psychiatric outpatients. Psychosomatics 50, 580–585. 10.1176/appi.psy.50.6.580 19996228

[B16] Heller D. P. Heller L. (2004). Somatic Experiencing® in the Treatment of Automobile Accident Trauma. U.S. Assoc. Body Psycho Ther. J. 3, 42–52.

[B17] Horn S. R. Charney D. S. Feder A. (2016). Understanding resilience: new approaches for preventing and treating PTSD. Exp. Neurol. 284, 119–132. 10.1016/j.expneurol.2016.07.002 27417856

[B18] Huffman M. C. (2016). Neuroendocrine and Psychological Factors Associated with Burnout, Compassion Fatigue, and Reduced Compassion Satisfaction in Mental Health Professionals. Doctoral dissertation, University of Nebraska at Omaha.

[B19] Johnson W. B. Bertschinger M. Snell A. K. Wilson A. (2014). Secondary trauma and ethical obligations for military psychologists: preserving compassion and competence in the crucible of combat. Psychol. Serv. 11, 68–74. 10.1037/a0033913 24564444

[B20] Kocalevent R. D. Hinz A. Brähler E. (2013). Standardization of a screening instrument (PHQ-15) for somatization syndromes in the general population. BMC Psychiatry 13:91. 10.1186/1471-244X-13-91 23514436 PMC3606198

[B21] Kroenke K. Spitzer R. Williams J. Lowe B. (2010a). The patient health questionnaire somatic, anxiety, and depressive symptom scales: a systematic review. Gen. Hosp. Psychiatry 32, 345–359. 10.1016/j.genhosppsych.2010.03.006 20633738

[B22] Kroenke K. Zhong X. Theobald D. Wu J. Tu W. Carpenter J. S. (2010b). Somatic symptoms in patients with cancer experiencing pain or depression: prevalence, disability, and health care use. Arch. Intern. Med. 170, 1686–1694. 10.1001/archinternmed.2010.337 20937930 PMC3174492

[B23] Kroll H. Macaulay T. (2016). A preliminary survey examining predictors of burnout in pain medicine physicians in the United States. Pain Phys. 19, E689–E696. 27389112

[B24] Leitch M. L. (2007). Somatic Experiencing® treatment with tsunami survivors in thailand: broadening the scope of early intervention. Traumatology 13, 11–20. 10.1177/1534765607305439

[B25] Leitch M. L. Vanslyke J. Allen M. (2009). Somatic Experiencing® treatment with social service workers following hurricanes katrina and rita. Soc. Work 54, 9–18. 10.1093/sw/54.1.9 19205253

[B26] Levine P. A. (2010). In an Unspoken Voice: How the Body Releases Trauma And Restores Goodness. Berkeley, CA: North Atlantic Books.

[B27] Li M. Y. Yang Y. L. Liu L. Wang L. (2016). Effects of social support, hope and resilience on quality of life among Chinese bladder cancer patients: a cross-sectional study. Health Qual. Life Outcomes 14:73. 10.1186/s12955-016-0481-z 27153944 PMC4859956

[B28] Ménard C. Pfau M. L. Hodes G. E. Russo S. J. (2016). Immune and neuroendocrine mechanisms of stress vulnerability and resilience. Neuropsychopharmacology 42, 62–80. 10.1038/npp.2016.90 27291462 PMC5143517

[B29] Nila K. Holt D. V. Ditzen B. Aguilar-Raab C. (2016). Mindfulness-based stress reduction (MBSR) enhances distress tolerance and resilience through changes in mindfulness. Ment. Health Prevent. 4, 36–41. 10.1016/j.mhp.2016.01.001

[B30] Park E. R. Traeger L. Vranceanu A. M. Scult M. Lerner J. A. Benson H.. (2013). The development of a patient-centered program based on the relaxation response: the Relaxation Response Resiliency Program (3RP). Psychosomatics 54, 165–174. 10.1016/j.psym.2012.09.001 23352048

[B31] Parker C. Doctor R. M. Selvam R. (2008). Somatic therapy treatment effects with tsunami survivors. Traumatology 14, 103–109. 10.1177/1534765608319080

[B32] Payne P. Levine P. A. Crane-Godreau M. A. (2015). Somatic experiencing: using interoception and proprioception as core elements of trauma therapy. Front. Psychol. 6:93 10.3389/fpsyg.2015.00093 25699005 PMC4316402

[B33] Pearlman L. Saakvitne K. (1995). Trauma and the therapist: countertransference and vicarious traumatization in psychotherapy with incest survivors. Am. J. Clin. Hyp. 4:451 10.1080/00029157.1996.10403354

[B34] Pereira J. A. Barkham M. Kellett S. Saxon D. (2016). The role of practitioner resilience and mindfulness in effective practice: a practice-based feasibility study. Adm. Policy Ment. Health Ment. Health Serv. Res. 44, 691–704. 10.1007/s10488-016-0747-0 27424107 PMC5550533

[B35] Power M. (1998). Development of the World Health Organization WHOQOL-BREF quality of life assessment. Psychol. Med. 28, 551–558. 10.1017/S0033291798006667 9626712

[B36] Richardson G. E. Waite P. J. (2002). Mental health promotion through resilience and resiliency education. Int. J. Emerg. Ment. Health 4, 65–75. 12014295

[B37] Sabin-Farrell R. Turpin G. (2003). Vicarious traumatization: implications for the mental health of health workers? Clin. Psychol. Rev. 23, 449–480. 10.1016/S0272-7358(03)00030-8 12729680

[B38] Shoji K. Cieslak R. Smoktunowicz E. Rogala A. Benight C. C. Luszczynska A. (2016). Associations between job burnout and self-efficacy: a meta-analysis. Anxiety Stress Coping 29, 367–386. 10.1080/10615806.2015.1058369 26080024

[B39] Sprang G. Clark J. J. Whitt-Woosley A. (2007). Compassion fatigue, compassion satisfaction, and burnout: factors impacting a professional's quality of life. J. Loss Trauma 12, 259–280. 10.1080/15325020701238093

[B40] The WHOQOL Group (1995). The World Health Organization quality of life assessment (WHOQOL): position paper from the world health organization. Soc. Sci. Med. 41, 1403–1409. 10.1016/0277-9536(95)00112-K 8560308

[B41] Thompson R. W. Arnkoff D. B. Glass C. R. (2011). Conceptualizing mindfulness and acceptance as components of psychological resilience to trauma. Trauma Viol. Abuse 12, 220–235. 10.1177/1524838011416375 21908440

[B42] Trippany R. L. Kress V. E. W. Wilcoxon S. A. (2004). Preventing vicarious trauma: what counselors should know when working with trauma survivors. J. Counsel. Dev. 82, 31–37. 10.1002/j.1556-6678.2004.tb00283.x

[B43] Tugade M. M. Fredrickson B. L. (2004). Resilient individuals use positive emotions to bounce back from negative emotional experiences. J. Pers. Soc. Psychol. 86:320. 10.1037/0022-3514.86.2.320 14769087 PMC3132556

[B44] van der Kolk B. A. (1984). Post-Traumatic Stress Disorder: Psychological and Biological Sequelae. Washington, DC: American Psychiatric Pub Inc.

[B45] Whitehouse B. Heller D. (2008). Heart Rate in trauma: patterns found in somatic experiencing and trauma resolution. Biofeedback 1, 24–29.

